# Quantification of Tissue Compression Identifies High-Grade Glioma Patients with Reduced Survival

**DOI:** 10.3390/cancers14071725

**Published:** 2022-03-28

**Authors:** Elies Fuster-Garcia, Ivar Thokle Hovden, Siri Fløgstad Svensson, Christopher Larsson, Jonas Vardal, Atle Bjørnerud, Kyrre E. Emblem

**Affiliations:** 1 Biomedical Data Science Laboratory, Instituto Universitario de Tecnologías de la Información y Comunicaciones, Universitat Politècnica de València, 46022 Valencia, Spain; 2 Department of Diagnostic Physics, Oslo University Hospital, 0372 Oslo, Norway; ivarth@student.matnat.uio.no (I.T.H.); s.f.svensson@fys.uio.no (S.F.S.); kyrre.eeg.emblem@rr-research.no (K.E.E.); 3 Department of Physics, University of Oslo, 0316 Oslo, Norway; atle.bjornerud@fys.uio.no; 4 Department of Neurosurgery, Oslo University Hospital, 0372 Oslo, Norway; larsch@ous-hf.no; 5 Unit for Computational Radiology and Artificial Intelligence, Oslo University Hospital, 0372 Oslo, Norway; jonvar@vestreviken.no; 6 Department of Radiology, Drammen Hospital, Vestre Viken Hospital Trust, 3004 Drammen, Norway; 7 Department of Psychology, Faculty for Social Sciences, University of Oslo, 0851 Oslo, Norway

**Keywords:** magnetic resonance imaging, high-grade glioma, longitudinal studies, compression, mass effect

## Abstract

**Simple Summary:**

A growing high-grade glioma exerts a local pressure on its surroundings, resulting in a tissue displacement known as the gross mass effect that is considered a major cause of acute neurological symptoms in patients with brain cancer. Mass effects are usually manifested when significant deformations caused by the tumor growth are observed radiologically or clinically; however, minor deformations in peritumoral tissue could provide early evidence of processes related to tumor relapse and recurrence. In this study, we propose an automated method to quantify the subtle deformations that occur in the peritumoral region. We also propose four biomarkers for differentiating where peritumoral displacements translate into compression. Biomarkers quantifying peritumoral compression were found to be associated with patient progression and prognosis and demonstrated the ability to stratify patients between long-time and short-time survivors. We conclude that compression biomarkers can be key to early treatment assessment during follow-up.

**Abstract:**

The compression of peritumoral healthy tissue in brain tumor patients is considered a major cause of the life-threatening neurologic symptoms. Although significant deformations caused by the tumor growth can be observed radiologically, the quantification of minor tissue deformations have not been widely investigated. In this study, we propose a method to quantify subtle peritumoral deformations. A total of 127 MRI longitudinal studies from 23 patients with high-grade glioma were included. We estimate longitudinal displacement fields based on a symmetric normalization algorithm and we propose four biomarkers. We assess the interpatient and intrapatient association between proposed biomarkers and the survival based on Cox analyses, and the potential of the biomarkers to stratify patients according to their survival based on Kaplan–Meier analysis. Biomarkers show a significant intrapatient association with survival (*p* < 0.05); however, only compression biomarkers show the ability to stratify patients between those with higher and lower overall survival (AUC = 0.83, HR = 6.30, *p* < 0.05 for CompCH). The compression biomarkers present three times higher Hazard Ratios than those representing only displacement. Our study provides a robust and automated method for quantifying and delineating compression in the peritumoral area. Based on the proposed methodology, we found an association between lower compression in the peritumoral area and good prognosis in high-grade glial tumors.

## 1. Introduction

High-grade brain tumors in adults are characterized by a highly infiltrative nature, cellular heterogeneity, and angiogenesis [1]. Despite advances in treatment modalities, high-grade glioma remains an incurable clinical challenge in which patient overall survival has not substantially improved in the last 20 years [2]. 

Owing to cancer cell proliferation and remodeling of the microenvironment [3], a growing high-grade glioma exerts a local pressure on its surroundings and results in a tissue displacement known as the gross mass effect. Mass effect is considered a major cause of acute neurological symptoms seen in patients with brain cancer [4], causing severe disability or even death, and it is a known prognostic factor for high-grade glioma [5,6,7]. Because the space occupied by the brain is restricted by the cranium, this pathological growth not only implies displacement, but also compression of the surrounding tissue [8]. The compression of peritumoral healthy tissue directly impacts the neurological function of the brain, psychological health, and patient quality of life [4].

Mass effects are usually manifested when significant deformations caused by the tumor growth is observed radiologically or clinically [5,9,10]. However, minor deformations in tissues close to the solid tumor mass have not been widely assessed and could provide early evidence of the processes related to tumor relapse and recurrence [11]. In vivo observations of structural displacements from tumor recurrence or growth are technically challenging, and are contingent on proper post-processing and interpretation tools. In a busy clinical workup, it is time consuming, and not technically feasible, for medical specialists to manually process longitudinal MRI exams for every single patient.

In this study, we propose an automated method for longitudinal image analysis that allows us to quantify and characterize the subtle deformations that occur in the peritumoral region. This method delineates subregions within peritumoral area that are most affected by compression phenomena (compression habitats) and quantify displacement and compression phenomena by defining four biomarkers. To study and compare the clinical relevance of these biomarkers, we assess the relationship between these biomarkers and progression status based on the RANO criteria. Moreover, we study the inter- and intrapatient association between the proposed biomarkers and the survival of high-grade glioma patients. Finally, we assess how tissue deformation may help stratify patients according to their overall survival. 

Biomarkers characterizing peritumoral compression were associated with patient progression (according to RANO criteria) and patient prognosis, and demonstrated the ability to stratify patients between long-time and short-time survivors. We consider that the proposed method based on the definition of compression habitats and the quantification of the associated phenomena could provide a relevant tool for early progression assessment as well as provide key enabling information to improve monitoring of high-grade glioma patients.

## 2. Materials and Methods

### 2.1. Patient Population

Two-hundred and twenty nine MRI exams from 27 patients with histologically confirmed high-grade glioma treated at our institution were eligible for inclusion in this study [12]. Among the 27 patients, 24 were originally diagnosed as glioblastoma (3 with IDH mutation, 2 with wild-type IDH mutation, 19 with unknown IDH mutation status) based on the 2016 WHO Classification of Tumors of the Central Nervous System. The remaining 3 were diagnosed as 1 anaplastic oligodendroglioma and 2 anaplastic astrocytoma. All patients provided written informed consent before imaging and following approval from the regional ethics committee. Treatment was based on the standard protocol for adult patients with high-grade glioma as proposed by Stupp et al. [13], including surgery, followed by stereotactic radiotherapy approximately four weeks after surgery with concomitant and adjuvant chemotherapy with temozolomide for a minimum of 6 weeks. Imaging was performed immediately before the start of radio-chemotherapy, every second week during this treatment, as well as two weeks after treatment. Imaging was then performed 2, 3, 6, and 12 months after chemotherapy initiation, and biannually afterward, until there was evidence of clinical deterioration, neurological deterioration, or death. Radiographic progression-free survival was defined as time to progressive disease according to the updated RANO criteria [14]. Three neuroradiologists (4–22 years of experience) made a consensus agreement for each patient case. 

Of the 27 patients in the original cohort, 80 MR exams performed less than 30 days after the previous exam were excluded to ensure the quality in estimating deformation fields. As a result, 127 MRI longitudinal studies from 23 patients were finally included.

### 2.2. MRI and Lesion Segmentations

The MRI exams were performed on a 3 Tesla Philips Achieva (Philips Medical Systems, Best, The Netherlands), using an eight-channel head coil. Structural imaging included a 3D FLAIR (echo time (TE)/repetition time (TR)/inversion time (TI) (ms) = 424/8000/2400, voxel size 1.07 × 1.07 × 0.6 mm^3^, matrix 224 × 224, 300 slices) and a 3D T1-weighted gradient echo before and after contrast agent injection (T1-CE, TE/TR = 2.3/5.1 ms, voxel size 1 × 1 × 1 mm^3^, matrix 256 × 232, 190 slices). 

Contrast-enhanced tumor and edema regions were annotated using a semi-automatic method previously described [12]. These ROIs were edited and approved by a radiologist (4 years of experience). 

### 2.3. Biomarkers

**Figure 1:**
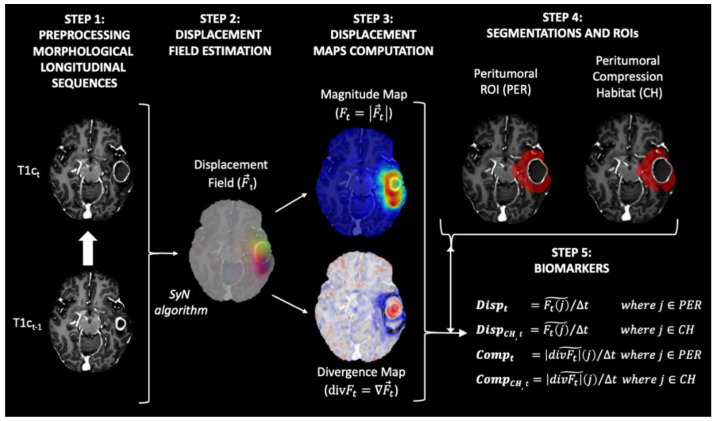
Diagram of the proposed method for obtaining the biomarkers proposed in our study. All maps and masks were superimposed on the T1-weighted contrast-enhanced image obtained at time t, with the exception of the maps in Step 3. In this step, the T1-weighted contrast-enhanced image obtained at time t−1 was used to improve interpretability.

In this study, we propose a methodology for the estimation of biomarkers consisting of the following steps: (1) image preprocessing of each MRI exam, (2) longitudinal intrapatient registration and displacement field estimation, (3) computation of displacement and divergence maps, (4) delineation of peritumoral ROI and identification of compression habitats, and (5) computation of biomarkers (see Figure 1).

Step 1 Image preprocessing of each MRI exam

Preprocessing of the structural MRI data was based on the ONCOhabitats pipeline defined in [15] and ANTs suite [16], and included the following steps: (a) voxel isotropic resampling to 1 × 1 × 1 mm^3^ of all MR images using a linear interpolation, (b) denoising based on the adaptive non-local means filter, (c) rigid intrapatient registration between the different sequences, (d) affine registration to MNI space, (e) skull stripping based on convolutional neural networks, and (f) magnetic field inhomogeneity correction based on N4 algorithm.

Step 2 Longitudinal interpatient registration and displacement field estimation

All MRI exams for each patient were registered longitudinally to the patient’s first longitudinal MR exam, which was used as reference. To do so, we used both rigid and affine transformations, with cross correlation as an optimization metric. After that we computed the displacement field between each contrast-enhanced T1-weighted (T1c) image and the corresponding T1c image of the previous exam. To compute the displacement field, a symmetric normalization (SyN) algorithm [16] implemented in the antsRegistration function of the ANTs suite [17] was used. The parameters used to compute the displacement field were: (1) metrics: ANTS neighborhood cross correlation; (2) transform type: SyN (gradient Step: 0.1, update Field Variance In Voxel Space = 3, total Field Variance in Voxel Space = 0); (3) convergence (iterations per level = 100 × 70 × 50 × 20, convergence Threshold = 1 × 10^−^^6^ convergence Window Size = 10; (4) shrink factors at each level: 8 × 4 × 2 × 1; (5) sigma of Gaussian smoothing at each level: 3 × 2 × 1 × 0 voxels.

The resulting displacement field represents the displacement in the three directions x, y, and z applied to each voxel to match each T1c image with their corresponding T1c image of the previous exam (see Figure 1, step 2).

Step 3 Computation of displacement and divergence maps

To transform the deformation fields into scalar maps, the magnitude ($F_{t})$ and the divergence maps ($\mathrm{div}F_{t})$ were calculated from the deformation field (${\vec{F}}_{t}$) (see Figure 1, step 2) as follows:

Magnitude Map $F_{t}=\left|{\vec{F}}_{t}\right|$.

Divergence Map $\mathrm{div}F_{t}=\nabla {\vec{F}}_{t}$.

The magnitude map shows how much displacement is occurring around each voxel. In contrast, the divergence map shows the degree to which the tissue is expanding (positive divergence) or contracting (negative divergence) around each voxel.

Step 4 Delineation peritumoral ROI and identification of compression habitats

The region most affected by the mass effect produced by tumor growth is the one closest to the active tumor. This peritumoral region for each exam was defined as the segmented tumor core mask (i.e., enhancing tumor + necrosis + postsurgical cavities) obtained from the last image exam available for each patient and dilated by 2 cm, minus the tumor core mask at the current exam (see Figure 1, step 4). 

In addition, we aim to assess regions where tissue displacement leads to tissue compression. For this purpose, we defined the compression habitats as the regions within the peritumoral ROI that showed a contractive behavior (i.e., present negative values in the divergence map).

Step 5 Computation of biomarkers

We propose four biomarkers to summarize the displacement and compression assessments in the peritumoral region for each MRI study:

- Displacement (Disp): median value of the magnitude map ($F_{t}$) in the peritumoral ROI.
- Displacement in the compression habitat (Disp_CH_): median value of the magnitude map ($F_{t}$) in the peritumoral compression habitat.
- Compression (Comp): median absolute value of the divergence map $(divF_{t}$) in the peritumoral ROI.
- Compression in the compression habitat (Comp_CH_): median absolute value of the divergence map ($divF_{t}$) in the peritumoral compression habitat.

To avoid biases due to different time intervals between MRI exams, all biomarkers were normalized by the time between the exams at timepoints t−1 and t (see Figure 1, step 1). This time between examinations is expressed in 90-day periods. In this way, the displacement values shown in mm represent the displacement caused by tumor growth during a typical follow-up period.

### 2.4. Statistical Analysis

We first analyzed whether the proposed biomarkers were related to the tumor progression status estimated by the RANO criteria. To do so, we compared the median values of biomarkers between progression and non-progression (including partial response, pseudo-response, and stable status) status examinations for each patient. In this way, we avoided the potential introduction of bias into the results due to the different numbers of MRI studies available for each patient. To assess the differences, we used the non-parametric Wilcoxon signed-rank test with a significance level of *p* < 0.05. Additionally, we repeated the analysis comparing all pairs of MRI studies (progressing vs. non-progressing) for each patient, instead of just the median, in order to visualize the patterns using all available data. 

To analyze interpatient association between biomarkers and patient overall survival, we used uniparametric Cox Proportional-Hazards regression analyses. The biomarker value for each patient was defined as the median of all the longitudinal values available for the patient. To analyze intrapatient association between biomarkers and time-to-exitus (defined as the time from each MRI study to exitus), we used multiparametric Cox Proportional-Hazards regression analyses. To eliminate the dependency on each patient we included as binary co-variables whether each sample (i.e., biomarker value) belonged to each patient. 

Additionally, we performed a Kaplan–Meier survival analysis with log-rank tests to assess differences in overall survival between patients divided by biomarker thresholds. Stratification thresholds for each biomarker were defined as those which best separated populations in terms of their C-index [18]. To avoid influence of non-representative subsets, we always ensured that the size of subpopulations was greater than 25% of the total number of cases. For censored cases, we set the date of censorship to the last date of contact with the patient or, in cases where this information was not available, the date of the last MRI exam. 

All *p*-values were adjusted by false discovery rate (FDR) using the Benjamin and Hochberg procedure [19].

## 3. Results

**Figure 2:**
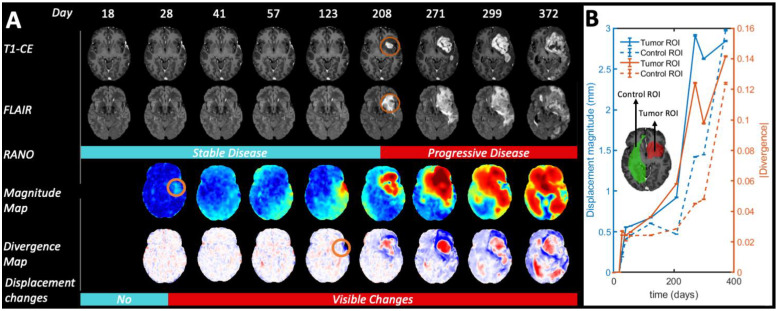
Longitudinal evolution of Patient 4 from day 18 after start of radio-chemotherapy treatment to day 372. For illustration purposes, we did not exclude the first MRI exams with periods shorter 30 days from the previous one in this figure, as described in the inclusion criteria for the rest of the statistical analysis. (**A**) According to RANO criteria, tumor progression started on day 208. However, the displacement maps show significant deformations already at day 28. Circles in orange highlight preliminary visual evidence of tumor growth. (**B**) Quantification of the displacement magnitude and absolute divergence in control and tumor ROIs, respectively.

In Figure 2A, a full longitudinal study of a patient with high-grade glioma is presented to illustrate how the magnitude maps and divergence maps characterize tumor evolution during follow-up. For this patient, changes in the divergence and magnitude maps are observed earlier than changes by traditional RANO criteria of recurrent hyperintensity in the T1-CE images as identified by the expert radiologist (see Figure 2B). 

### 3.1. Association with Tumor Progression Based on RANO Criteria

The non-parametric Wilcoxon signed-rank paired test on the differences between median biomarkers in progressing versus non-progressing paired exams from each patient showed that all biomarkers (Disp, Disp_CH_, Comp_,_ Comp_CH_) showed significantly higher values in MRI studies labeled as progressing compared those labeled as non-progressing for each patient (*p* < 0.05). These differences are more evident in the compression biomarkers (Comp, Comp_CH_) than in the displacement biomarkers (Disp, Disp_CH_).

### 3.2. Association with Patient Overall Survival

**Figure 3:**
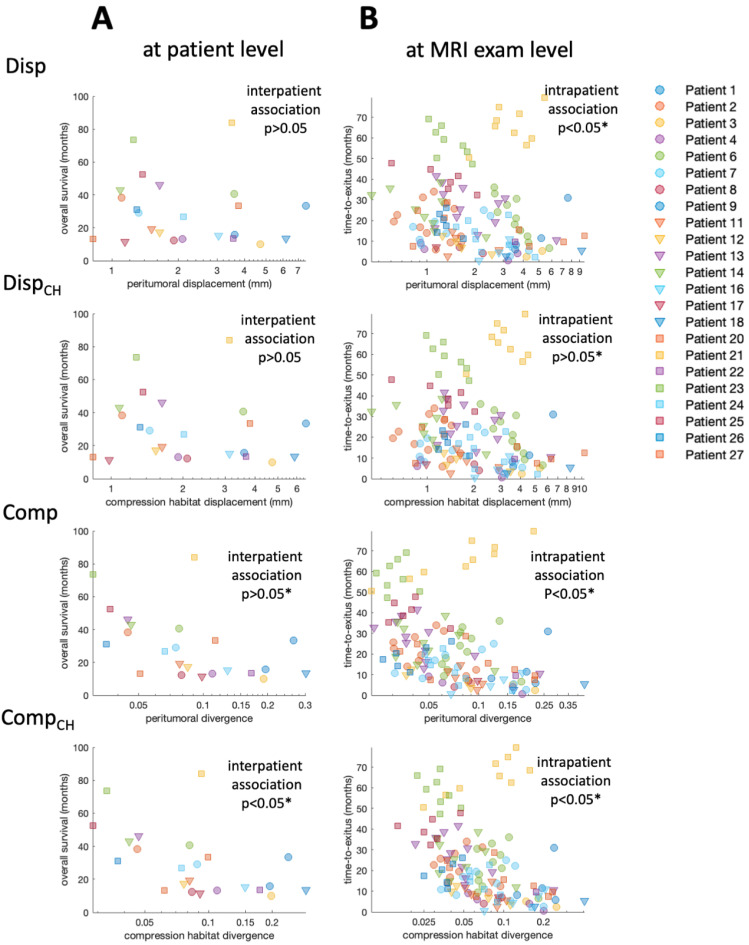
(**A**) Scatter plot showing the relation between median biomarker for each patient and overall survival. (**B**) Scatter plot showing the relation between biomarker for each MRI study and time-to-exitus. Each combination of marker color and shape corresponds to a different patient included in the study. * Indicates significant difference (*p* < 0.05).

The interpatient association between the median biomarkers and patient overall survival is presented visually in the log-plot of Figure 3A. In this figure, an inverse relationship between the values of the four biomarkers and patient overall survival is observed. Figure 3B includes not only the mean values of the biomarkers for each patient, but also the values obtained in each of the longitudinal MRI studies carried out during the patient follow-up. In contrast to Figure 3A, in Figure 3B, the concept of overall survival is replaced with time-to-exitus. The different biomarkers’ colors and shapes represent data from different patients (see legend). 

Results of Cox regression for both interpatient and intrapatient associations between biomarkers and patient survival are presented in Table 1. The interpatient association between biomarkers and overall patient survival is visible, especially for the compression biomarkers (Figure 3A). However, due to the low number of cases, the Cox analysis only shows a significant association for Comp and Comp_CH_ before correcting *p*-value for FDR. The intrapatient association between biomarkers and time-to-exitus assessed by Cox analysis is significant (*p* < 0.05) for all biomarkers, even after correction for FDR. Biomarker Hazard Ratios are higher when they are calculated in the peritumoral compression habitat. In addition, Hazard Ratios are higher for compression biomarkers than for displacement biomarkers.

### 3.3. Stratification Capability

**Figure 4:**
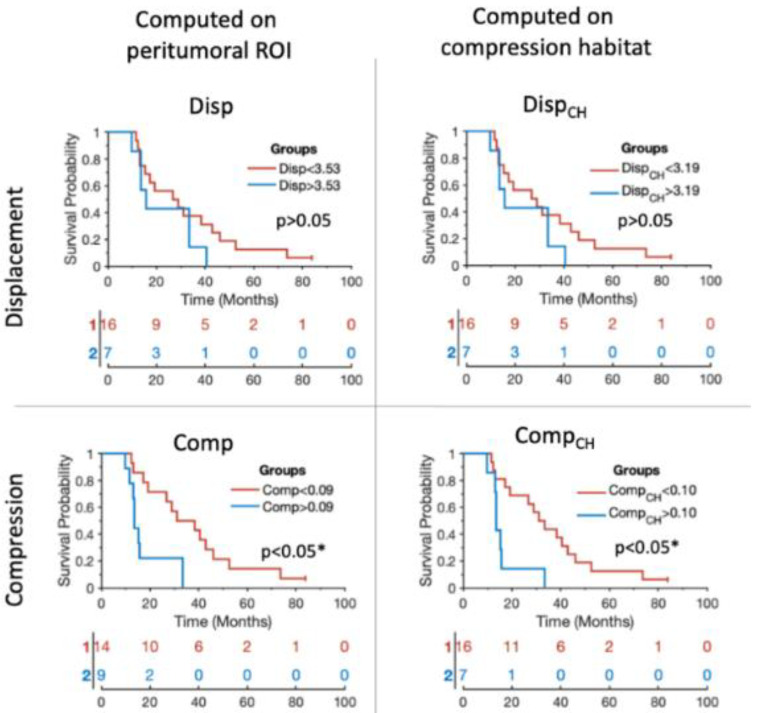
Kaplan–Meier plots showing the stratification capability of the median biomarkers proposed for each of the patients included in the study. Blue lines represent the patients showing higher tumor mass effect according to each of the biomarkers. Red lines represent the patients showing lower tumor mass effect according to each of the biomarkers. The x axes represent the overall survival in months. * Indicates significant difference (*p* < 0.05).

The ability to stratify patients between those with higher and lower overall survival based on biomarkers is also observed to be significant (*p* < 0.05) for compression biomarkers (see Table 2 and Figure 4). Stratification based on those compression biomarkers (i.e., Comp and Comp_CH_) obtains high AUC values (AUC = 0.82 and AUC = 0.83, respectively). Similarly to the results obtained in the Cox analysis, the stratification results improve when using compression biomarkers, and when the biomarkers are calculated from the values within the compression habitat.

## 4. Discussion

The compression of peritumoral healthy tissue in brain tumor patients is considered a major cause of life-threatening neurologic symptoms [4]. Gross mass effect is usually assessed qualitatively by the treating physician, and only taken into consideration in the later stages of the disease when the deformation caused by the tumor growth is apparent and advanced. Numerous studies in the literature confirm the prognostic ability of the mass effect produced by tumor growth [5,6,20,21]. However, there are fewer studies dedicated to the quantification of these tissue displacements, and most of them are focused on the more macroscopic phenomena such as midline shift [9,10] or in the displacement of the lateral ventricles [5]. On the contrary, the quantification of minor tissue deformations and their associated compression has not been widely investigated. 

In this work we proposed a methodology to automatically quantify small displacements from tumor growth. We use the information provided by nonlinear registration based on symmetric normalization algorithm to estimate the displacement field. Unlike previous work [11], we estimate the displacements with respect to a series of longitudinal MRI studies and not by a standard atlas. This allows us to monitor tumor evolution during patient follow-up. Moreover, we propose a method to characterize when and where these displacements translate into compression of tissues near the tumor (compression habitats) based on the estimation of the divergence of the displacement field. Although displacement and compression are associated phenomena, displacement observed in a region does not always imply compression in the same region. Compression in eloquent areas [22], and not just the displacement, may constitute a major impact on neurological function. 

In this study, we first assessed whether the proposed biomarkers differed significantly with the progression versus non-progression status of each patient. The results show that all biomarkers had significantly higher values when the tumor was progressing than when the tumor was not progressing. In particular, the biomarkers characterizing tissue compression were differentiated between progressing and non-progressing tumor. This higher performance of the compression biomarkers may be because the divergence operation (in the basis of quantification of compression) could be more robust to suboptimal intrapatient registrations during preprocessing. That is, intrapatient rigid registration errors during preprocessing could generate a constant bias in the displacement field. This bias affects the quantification of the displacement biomarkers (as they are based on the magnitude of the displacement field), but not by the compression biomarkers (as they are based on divergence operator). 

To assess the clinical relevance of the proposed biomarkers, we investigated the interpatient association between proposed biomarkers and the overall survival of each patient. To avoid introducing any bias by not having the same number of MRI scans during follow-up in each patient, the interpatient association analysis based on Cox regression was done using only one value for each patient (i.e., the median of the biomarkers over the entire follow-up). This conservative approach, together with the FDR correction, make this association non-significant in the Cox analysis. The visualization of the data shows a clear pattern between the median compression biomarkers and the overall survival. This pattern is consistent, and is even more evident if we do not use just the median value per patient, but use all available values obtained in each MRI exam acquired during the follow-up. We also analyzed the intrapatient association between biomarkers and time-to-exitus. This intrapatient association assessed by multiparametric Cox analysis is significant for all biomarkers. Analysis of the Hazard Ratios suggests that estimating biomarkers calculated in the compressed habitat rather than in the entire peritumoral area could improve the performance of these biomarkers. These results show that most of the proposed biomarkers could be relevant for monitoring the patient’s evolution during follow-up and help in the estimation of prognosis. Finally, we analyzed whether the proposed biomarkers could be useful for stratifying patients according to their overall survival. The results obtained show that biomarkers based on compression characterization (i.e., Comp and Comp_CH_) are able to divide the population of high and low survivors obtaining high AUC values, a substantial difference between the mean survival of both groups, and elevated Hazard Ratios. These results indicate that compression biomarkers show a stronger association with overall survival of high-grade glioma patients (interpatient variability) while displacement-based biomarkers are slightly more relevant for the study of intrapatient evolution. 

Our results indicate that compression of peritumoral tissue due to tumor growth is associated with poor patient prognosis. A general trend indicates that both displacement and compression biomarkers improve their association with patient prognosis when estimated in the compression habitat. Based on these results, we consider the identification of compression habitats in the peritumoral area to improve the robustness of the biomarkers and provide valuable information to predict the effects of tumor growth. In addition, these results may indicate that compression habitats would be particularly relevant areas to examine during patient follow-up. 

The methods presented in this study could be used to assess in humans the causal link between solid stress and neurological dysfunction found in recent preclinical studies [4]. Future work should use the proposed methodology and biomarkers to assess the influence of tissue compression near eloquent areas and its subsequent impact on neurological function. Additionally, future work should analyze the associations between the compression habitats and measures of mechanical stress in these regions, as obtained by magnetic resonance elastography [23,24,25]. This would allow us to validate the interpretation of the proposed habitats and to assess to what extent the information provided by both techniques are complementary for longitudinal monitoring of patients with high-grade gliomas. Finally, future work should evaluate the different levels of ability of the proposed markers for the assessment of early progression in compressive and infiltrative tumor phenotypes.

The relatively low number of patients included in the analysis represents a limitation of our study. However, a large number of time points (i.e., 127 MRI studies) were available for the 23 patients included. While logistically demanding, this setup allowed us to overcome the limitations of the few follow-up MRI exams that make out standard clinical diagnostic procedures. Therefore, our study provides a theoretical basis for the proposed biomarkers to be adapted in larger clinical cohorts where the number of available longitudinal examinations is restricted. Another limitation of the study is that it is hypothesized that the main contributor to tissue compression is tumor growth; nevertheless, other processes such as cystic changes, inflammation, the effects of radiotherapy, tissue relaxation after surgery, or ventricular expansion may also contribute to the observed effect. These phenomena could also contribute significantly to the value of biomarkers and should be taken into consideration in their interpretation. In terms of the whole cohort, and despite all these confounding factors, we observed an association of the proposed biomarkers and clinical endpoints related to survival. Finally, the biomarkers proposed in this study show associations with patient survival; however, further work with larger cohorts and an independent test set are needed to confirm the possible prognostic capabilities of these markers and their ability to show the tendency for earlier recurrence.

## 5. Conclusions

In summary, our study provides a robust and automated quantification of compression in the peritumoral area and a methodology to assess the areas most affected by this phenomenon. Based on our proposed methodology, we found a significant association between lower compression in the peritumoral area and good prognosis for the patient. Future validation of our findings in multicenter cohorts may make this method a tool with the potential to improve the follow-up of patients with high-grade glioma. 

## Figures and Tables

**Table 1:** Results for the Cox regression analyses and their associations with patient prognostic. The interpatient association analysis shows the results of the uniparametric Cox regression for biomarkers to predict OS. The interpatient association analysis shows the results of the multiparametric Cox regression for biomarkers to predict OS. To eliminate the dependency on each patient, we included whether each biomarker value belonged to each patient as a binary co-variable. Asterisk * indicates significant difference (*p* < 0.05).

**INTERPATIENT ASSOCIATION (*n* = 23 Patients)**				
	**Hazard Ratio** **[95% Conf. Interval]**	**Hazard Ratio** **[95% Conf. Interval]** **Normalized Var.**	***p*-Value**	***p*-Value (FDR Adjusted)**
Disp	1.05 [0.83, 1.34]	1.43 [0.28, 7.37]	0.666	0.666
Disp_CH_	1.09 [0.83, 1.44]	1.65 [0.35, 7.86]	0.527	0.666
Comp	250.27 [1.04, 6.02 × 10^4^]	4.45 [1.01, 19.58]	0.048 *	0.097
Comp_CH_	829.55 [2.15, 3.19 × 10^5^]	5.75 [1.22, 27.10]	0.027 *	0.097
**INTRAPATIENT ASSOCIATION (*n* = 127 MRI Exams)**				
	**Hazard Ratio** **[95% Conf. Interval]**	**Hazard Ratio** **[95% Conf. Interval]** **Normalized Var.**	***p*** **-Value**	***p*** **-Value (FDR Adjusted)**
Disp	1.43 [1.20, 1.70]	26.07 [5.32, 127.83]	5.83 × 10^−5^ *	1.19 × 10^−4^ *
Disp_CH_	1.39 [1.19, 1.64]	27.41 [5.44, 138.01]	5.93 × 10^−5^ *	1.19 × 10^−4^ *
Comp	3.72 × 10^4^ [44.87, 3.08 × 10^7^]	79.46 [4.86, 1.30 × 10^3^]	0.0021 *	0.0021 *
Comp_CH_	7.86 × 10^4^ [1.10 × 10^2^, 5.60 × 10^7^]	81.40 [6.26, 1.06 × 10^3^]	7.72 × 10^−4^ *	0.0010 *

**Table 2:** Results of the log-rank test of the Kaplan–Meier analysis. For each biomarker, the median OS and number of patients with high and low biomarker value are presented. Additionally, differences between OS (months), hazard ratios, area under the curve (AUC), and log-rank test resulting *p*-value are presented. * Indicates significant difference (*p* < 0.05).

	Cut-Off Threshold	Patients per Group [Low, High]	AUC (C-Index)	Median OS per Group [Low, High]	Hazard Ratio [95% Conf. Interval]	*p*-Value (Log-Rank Test)	*p*-Value (FDR Adjusted)
Disp	3.53	[16, 7]	0.73	[27, 16]	1.86 [0.65, 5.34]	0.250	0.250
Disp_CH_	3.19	[16, 7]	0.74	[27, 16]	1.86 [0.65, 5.34]	0.250	0.250
Comp	0.09	[14, 9]	0.82	[31, 14]	5.33 [1.69, 16.80]	0.004 *	0.012 *
Comp_CH_	0.10	[16, 7]	0.83	[31, 14]	6.30 [1.69, 23.42]	0.006 *	0.012 *

## Data Availability

The data presented in this study is available on request from the corresponding author.
